# Falls Prevention and Quality of Life Improvement by Square Stepping Exercise in People with Parkinson’s Disease: Project Report

**DOI:** 10.3390/jpm11050361

**Published:** 2021-04-30

**Authors:** Asunción Mayoral-Moreno, Carlos Alexis Chimpén-López, Laura Rodríguez-Santos, María Isabel Ramos-Fuentes, Francisco José Vaz-Leal, Manuel Alfredo Moral, Jorge Pérez-Gómez, José Carmelo Adsuar

**Affiliations:** 1 Psychiatry Area, Faculty of Nursing and Occupational Therapy, University of Extremadura, 10003 Cáceres, Spain; mmayoralm@unex.es; 2 Psychiatry Area, Faculty of Medicine and Health Sciences, University of Extremadura, 06006 Badajoz, Spain; laura@unex.es (L.R.-S.); miramos@unex.es (M.I.R.-F.); fjvaz@unex.es (F.J.V.-L.); 3 Department of Religion, Oakwood University, Huntsville, AL 35896, USA; manuelalfredomoral@gmail.com; 4 HEME Research Group, Faculty of Sport Science, University of Extremadura, 10003 Cáceres, Spain; jorgepg100@gmail.com (J.P.-G.); jadssal@unex.es (J.C.A.)

**Keywords:** anticipatory cognition, balance, cognitive aspects, depression, perceived social support

## Abstract

Parkinson’s disease (PD) is a chronic neurodegenerative disorder that affects physical, psychological, and social quality of life. Square Stepping Exercise (SSE) is an effective balance training program to prevent falls and to stimulate cognitive function in the elderly; however, no study has analyzed the effect of SSE in people with PD. The main objective is to investigate whether the application of SSE is safe, applicable, and can improve balance, and is effective in preventing falls, improving cognitive and psychological aspects and thus maximize quality of life in people with PD. Methods/Design: SSE will be performed three times per week for 8 weeks with an additional month follow-up after the intervention. Sixty people with PD will participate, randomly distributed into two groups: experimental group (SSE: *n* = 30) and control group (Usual care: *n* = 30). The primary measurements will be: (1) Applicability, (2) Safety, (3) Balance, and (4) Annual number of falls. Secondary measurements will be: (1) Sociodemographic information, (2) Physical condition, (3) Health-related quality of life, (4) Depressive symptoms, (5) Cognitive aspects, (6) Perceived functional social support, and (7) Anticipatory cognition.

## 1. Background

Parkinson’s disease (PD) is a neurodegenerative process that affects the nervous system in a chronic and progressive manner. It is mainly characterized by patients presenting a series of motor symptoms such as slowness of movement, resting tremor, muscle rigidity, or postural instability; there are also non-motor symptoms such as autonomic dysfunction, cognitive/neurobehavioral disorders, and sensory and sleep abnormalities [1]. It is the second most common neurodegenerative disease after Alzheimer’s disease [2]. In 2016, it was estimated that 6.1 million people worldwide have been diagnosed with PD, which represents an increase of more than double compared to the 2.5 million observed in 1990. By 2040, it is estimated that there will be around 17 million people affected [3]. This exponential increase in PD represents a greater social, economic, and health burden for public institutions and society. Therefore, it is necessary to implement new rehabilitative therapies that promote health, prevent falls, and maximize health-related quality of life (HRQoL) in people with PD.

HRQoL in PD is determined by motor and non-motor symptoms [4]. As the disease progresses, patients are more prone to HRQoL deterioration as a result of increased motor disability and non-motor symptom burden [5]. Among non-motor symptoms, depression has been recognized as the main predictor of low HRQoL [6,7] through causing a negative impact on activities of daily living and cognitive performance [8]. Depression is a prevalent mood disturbance in PD, affecting 2.7% to 90% of patients [9]. Other common symptoms in PD patients that affect HRQoL are anxiety and apathy [10]. In fact, studies have shown that depression often coexists with anxiety [11] and apathy [12]. Clinical presentations of depression share some similarities with anxiety and apathy, such as fatigue, agitation, psychomotor retardation, and difficulty concentrating [13]. In addition, apathy and depression can affect motivation to perform social functions and correlates with social isolation in PD [14].

Cognitive impairments are also part of the clinical symptomatology of PD, as patients’ cognitive ability gradually deteriorates during disease progression [15]. Executive function deficits are usually the most predominant (deficits in planning, abstract reasoning, and verbal fluency), along with memory deficits and visual-spatial deficits [16]. Similarly, cognitive impairments have an emotional impact on patients, which adds to the burden of physical symptoms of PD [17].

Anticipatory cognitions (AC) refer to individuals ability to formulate life prognoses as an attitude characterized by an intention to understand the future [18] and may enable or hinder the individuals in achieving the competence required for their goals [19]. In the disease process, the positive or negative meaning of these cognitions will affect expectations of the disease [20], allowing the prediction of future behaviors [21,22].

ACs would play a relevant role in chronic diseases [19], and their assessment could be useful in predicting treatment response and influencing overall treatment outcomes.

Among motor symptoms, postural instability and postural disturbances are the main determinants of HRQoL, as they are associated with increased disability and increased risk of falls [23]. Falls are a common problem in PD and can lead to fear of falls, injuries, hospitalizations, and hip fractures [24,25]. Falls represent a major health problem due to their high prevalence and the severity of their physical, functional, psychological, and economic consequences. Each year, about 50 million falls occur in Europe among older people, and the healthcare expenditure for the treatment of fall-related injuries in the European Union is estimated to be 25 billion euros per year [26]. The estimated prevalence of falls in PD varies from 48% to 72% and increases with advancing disease [27,28,29]. People with PD are three times more likely to suffer a hip fracture as a result of a fall compared to people without PD [30]. However, the severity of disability and fall rates can be reduced by physical exercise interventions [31]. Exercise has been recommended for patients with PD, regardless of their health status or the course of their disease [32]. Several studies evidence the effectiveness of performing physical exercise to improve motor impairment, activities of daily living, and quality of life in PD [33]. Physical activity may be an appropriate non-pharmacological therapy in PD. Non-motor symptoms, depression, apathy, fatigue, cognition, and sleep are significantly improved with physical exercise [34].

The Square Stepping Exercise (SSE) was created by Shigematsu and Okura in order to improve balance, thus decreasing the risk of falls [35]. It is a training program that requires physical exertion and cognitive function, specifically focusing on attention, memory, and executive functions [36,37]. The SSE comprises multiple steps in various directions, performed on a thin carpet that is divided into squares, 25 cm^2^ each [38]. It has even been observed that it is an apparently more effective alternative to walking for balance improvement, helping to reduce fall risk factors and being recommended as a health-promoting exercise in older people [39]. The few existing studies on SSE are promising and show the effectiveness of the program on the functional components of physical fitness, including balance, lower extremity strength, flexibility, and agility, therefore leading to a reduction in the risk of falls. Similarly, cognitive aspects are improved, particularly concentration, mental flexibility, and visual memory [40].

In order to have safety and applicability data in PD, long-term interventions can improve the knowledge in this issue [41]. This study presents the direct measures of safety and applicability of the SSE program, which offers evidence to implement physical exercise programs to PD.

The present study is developed with the aim of providing new rehabilitative therapies. The main objective is to investigate whether the application of SSE is safe, applicable, can improve balance, and is effective in preventing falls, improving cognitive and psychological aspects and thus maximizing quality of life in people with PD. In accordance with the stated objective, the main hypothesis of the study is that the SSE will be safe, applicable, and effective in improving balance and preventing falls and improving cognitive and psychological aspects and the health-related quality of life in people with PD.

## 2. Materials and Methods

### 2.1. Study Design

This study is a randomized controlled clinical trial and uses the Consolidated Standards for Reporting Trials (CONSORT) methodology for randomized controlled trials [42].

### 2.2. Ethics Approval

The project has obtained approval from the Bioethics and Biosafety Committee of the University of Extremadura (number 27/2020), from Cáceres, and the Drug Research Ethics Committee of the Department of Health and Social Services of the Regional Government of Extremadura (number 039-2002) and has been registered in the Clinical Trials Register provided by the Australian and New Zealand Clinical Trials Registry (application number 380493: https://www.anzctr.org.au/) (accessed date 15 October 2021).

### 2.3. Sample Size

The study population will be made up of patients with PD.

The *L*-test (a variant of the time up and go test), which has been shown to be valid and reliable in PD, was used for sample calculation. The sample calculation reflected that 60 people with PD are necessary (experimental group: *n* = 30 and control group: *n* = 30) for the study. Accepting an alpha risk of 0.05 and a beta risk of 0.2 in a two-sided test, 20 subjects are necessary in the first group and 20 in the second to recognize as statistically significant a difference greater than or equal to 4.35 units [36]. The common standard deviation is expected to be 5.6 [43] and the correlation coefficient between the initial and final measurement to be 0.7 [44]. The study anticipates a drop-out rate of 20%.

### 2.4. Randomization and Blinding

After the initial assessments, participants will be randomly assigned to either experimental (SSE) or control (“usual care”) groups. A simple computer-generated randomization sequence will be created prior to enrolling participants to assign participants to either the experimental or control group (1:1). The randomization sequence will be produced by a member of the research team with no clinical involvement in the trial. The allocation will be hidden in a password-protected computer file. Participants and the person in charge of the SSE sessions will know their group assignment; however, it will be hidden to the researchers. Data analysts will not be able to know the assignment.

### 2.5. Participants

Participants are to meet the following inclusion criteria:

- Be ≥18 years old.
- Have been diagnosed with PD by a neurologist, in stages I, II, or III, according to the Hoehn and Yahr scale [45].
- Not have pathology that contraindicates the physical exercise program (coronary pathologies, thrombosis, bone, kidney, moderate or severe pulmonary, severe psychiatric illness, etc.). The Physical Activity Fitness Questionnaire (PAR-Q) [22] will be administered to find out if they are living with diseases that prevent physical load.
- Not be suffering from moderate (those scoring 12–20) and severe cognitive impairment (<12), determined by Mini Mental State Examination scores [46].
- They must have signed the informed consent for the study.

### 2.6. Interventions

The participants will be distributed in two groups. Experimental group: They will participate in an SSE training program, three times a week for 8 weeks with a duration of 50 min for each session. Information on the protocol to be followed will be provided through a booklet, and each session will be supervised in person. The exercises will be performed by between 2 and 6 patients simultaneously in the same space, on a thin carpet with dimensions of 250 cm × 100 cm and divided into 40 smaller squares of 25 cm^2^. The execution will be performed at the rhythm given by a metronome. Rhythmic auditory stimulation is a neurological technique that uses the physiological effects of auditory rhythm on the motor system to improve rhythmic movements such as walking [47]. A pedometer will also be used to verify the volume of physical activity performed [38].

In the SSE there are 200 movement patterns that vary in difficulty and are classified into three categories: beginner, intermediate, and advanced. The beginner level has two levels in turn, while the intermediate and advanced levels have three levels each. The training progression is shown in Table 1. It will start with movement patterns similar to walking and gradually more complex patterns requiring not only forward, but also lateral, diagonal, and backward movements will be performed. Participants will be advised to avoid stepping on the dividing lines of the squares.

The SSE expert in each session will inform the participants about the exercise they have to perform and will show them the steps to follow in each class. The participants will also have a booklet with the 200 movement patterns, which they will be able to consult while the SSE expert shows them the steps to follow in each class. Each day, before starting the session, the last movement pattern performed in the previous class will be reviewed. Once the patients have memorized the pattern, which is expected to be achieved after about 4–5 repetitions based on a previous study [22,31,37], they will continue with the session autonomously.

Control group: Participants will continue with basic life activities and maintain the treatment established by the public health system (“usual care”). Although there is no standard treatment for PD, the usual treatment, depending on the clinical condition of each patient, consist of the prescription of medication, essentially Levodopa [48], and the recommendation of physiotherapy [49].

### 2.7. Measures and Procedures

The efficacy of the SSE will be evaluated by means of different questionnaires (Table 2).

#### 2.7.1. Primary Measurements

Applicability. This will be calculated as the percentage of participants with PD who are able to perform the set exercise. If any participant is unable to execute the intervention, the reason will be recorded [37].

Safety. In each session, any difficulties encountered, incidents, or injuries will be recorded, noting the possible cause of the problem [50].

Balance. This will be evaluated by the L-Test, which consists of measuring the time in which the participants stand up from a chair, walk 3 m, then turns 90 degrees, walk again 7 m, turn 180 degrees, and finally go back to the chair [43]. Additionally, the 3 m TUG [51] and 7 m TUG will be performed [52]. A familiarization test will be conducted. Time will begin to be measured when the evaluator gives the agreed command and will stop when the participant sits down resting his or her back against the back of the chair.

Number of falls. The number of falls suffered in the previous 6 months and last year will be recorded. A fall is defined as a sudden, involuntary change in position that causes a person to land on a lower level, i.e., on an object, the floor, or the ground, due to reasons other than sudden paralysis, epileptic seizures, or overwhelming external forces [53].

#### 2.7.2. Secondary Measurements

Sociodemographic data. A questionnaire will be filled in, focusing on data related to age, sex, marital status, educational level, work situation, living unit, time of diagnosis of the disease, non-pharmacological therapies, and frequency of falls in the previous 6 months and last year.

Fear of falling. Fear of falling will be measured using the Falls Efficacy Scale International (FES-I) questionnaire, developed and validated by the Prevention of Falls Network Europe. The questionnaire has excellent reliability and validity [54] in different cultures and languages [55], also in the Spanish version [56] and in PD [57]. It is a self-reported questionnaire that provides information on the level of concern about falls in a range of activities of daily living. The questionnaire collects 4 items for assessment (1 = not worried, 2 = somewhat worried, 3 = quite worried, 4 = very worried) and consists of 16 items.

Physical condition. Two-minute walking test. This measures the maximum distance, in meters, that each participant can walk in 2 min along a route [58].

Lower extremity strength. The test consists of counting the number of times the participant can stand up and sit on a chair for 30 s, in a seated position with the back straight and feet flat on the floor, without pushing off with the arms [59]. This test was applied in PD [60].

Speed. The “Brisk Walking Test” will be applied. This consists of measuring the time that each person uses to run 30 m. Two repetitions will be performed with a one-minute rest between them, and the best result will be recorded [61].

Functional Reach. The “Functional Reach Test” [62] will be used. The participant is positioned in profile next to a wall, and from that position is asked to flex the trunk, with the arms at 90 degrees, as far as possible without losing balance, remaining in that position for a few seconds. This test was also applied in PD [63].

Short Physical Performance Battery (SPPB). This comprises three direct observation tests, gait speed, balance, and time to get up five times from a chair [64].

Self-perception of physical fitness. The International Fitness Scale (International Fitness Scale-IFIS) [65] in the Spanish version [66] will be applied. This instrument allows us to know how the participants perceive their general physical condition, cardio-respiratory fitness, muscular strength, speed-agility, and flexibility. It consists of five questions and the response options are “very poor”, “poor”, “average”, “good”, and “very good”.

Cognitive aspects. The Brief International Cognitive Assessment (BICAMS) [67] will be administered, which focuses on cognitive processing speed and verbal and visual memory. BICAMS consists of three tests: the Symbol Digit Modalities Test (SDMT), which assesses processing speed/working memory; the California Verbal Learning Test (CVLT), which assesses verbal learning and memory; and the Brief Visual-spatial Memory Test (BVMT), which assesses visual-spatial learning and memory.

Psychological aspects:

- Health-related quality of life. This will be evaluated through the Parkinson’s Disease Questionnaire (PDQ)-8: This is a summarized index of only eight of the items present in the PDQ-39, which has proven to be valid and reliable in its English [68] and Spanish [69] versions, distributed in the following areas: mobility, activities of daily living, emotional, stigma, social support, cognition, communication, and bodily discomfort.
- Depression. The Beck Depression Inventory-II (BDI-II) [70], in its Spanish version [71], will be used. The BDI-II is a 21-item self-report instrument designed to assess the severity of depressive symptomatology. In each of its items, the person has to choose, from a set of four alternatives ordered from least to most severe, the statement that best describes his or her state during the last two weeks. Each item is valued from 0 to 3 points depending on the alternative chosen, and after directly adding the score of each item, a total score is obtained that varies from 0 to 63. The BDI-II is a test widely used to assess depression in chronic illness and in PD [72].
- Perceived Functional Social Support. The questionnaire (Duke-UNC) was designed to measure perceived functional social support by Broadhead [73], and it was validated for the Spanish population by Bellón (1996) [74]. It establishes 11 items and 5 response options for each of them: 1: Much less than I want, 2: Less than I want, 3: Neither much nor little, 4: Almost as much as I want, 5: As much as I want. Based on the total score obtained, support is considered normal for 32 or more points and low for less than 32 points.
- Anticipatory cognitions. The Anticipatory Cognitions Questionnaire (ACQ) of Légeron, Riviere, and Marboutin (1991), consists of eight questions on a Likert-type scale, with 4 response options assigning a score from 0 to 3 points ranging from true, almost always true, almost always false, or false options [75]. Items 1, 2, 4, 6, 7, and 8 refer to negative anticipatory cognitions, with the response “true” corresponding to 3 points and “false” to 0 points. Items 3 and 5 refer to positive anticipatory cognitions, corresponding the answer “true”, 0 points, and “false”, 3 points, the total score being the sum of the values assigned to all sections of the questionnaire with a total of 24 points; the cut-off point is set at 7 points.
  Familiarization and reliability tests. Before each physical test is measured, a trial test shall be carried out to familiarize each participant with each test and avoid the “learning effect”. A warm-up phase will be performed before the measurements. The participants will be informed about the development of the test prior to its execution. Reliability will be evaluated by repeating all the physical tests after one or two weeks with a sample of the participants. The reason for doing this is to know the smallest real difference of all the physical tests to be used in the study, due to the lack of information on this topic.

### 2.8. Statistical Analysis

The baseline characteristics of the participants will be presented as mean (standard deviation) for continuous variables and proportions for categorical variables. Two types of analysis will be performed:

Intention to treat analysis. This will include all randomly assigned participants in the analysis. Multiple imputation will be used to impute missing data. According to Barnes et al. [76], multiple imputation can be used with small sample clinical trials to avoid loss of information. The effects of the intervention on the primary and secondary variables will be assessed through repeated measures analyses of covariance adjusted for age and baseline values. The results will include the effect size (95% confidence interval) and statistical significance for each study measure with respect to time and its interaction effects (group × time). Statistical significance will be set at the conventional level of *p* < 0.05. In the sensitivity analyses, data imputation will be performed from the data of patients at baseline and those who completed the study, in order to avoid estimation biases.

Per-protocol analysis. Analyses similar to those described above will be performed, although only with those participants who have completed the SSE training program.

Testing several hypotheses increases the possibility of committing a type I error. To avoid this problem, the Bonferroni correction will be performed.

## 3. Discussion

The data obtained in this study will help us to clarify the applicability and safety of the SSE program in people with PD. To our knowledge, this will be the first study that provides this information.

The present study would be pioneering in investigating the effectiveness of SSE on fall prevention, improvement of cognitive and psychological aspects in people with PD, and its impact on HRQoL. In addition, it would be an innovative project that could perform a biopsychosocial assessment of PD patients by simultaneously addressing motor and non-motor symptoms.

Based on the described symptomatology of PD, it underlines the need to promote new programs to treat the disease; although the onset of neurodegenerative changes cannot be prevented, exercise can help in the rehabilitation of PD patients [77]. The results of several studies support that DFS [78] and physical exercise programs are effective in improving motor impairment, activities of daily living, and quality of life in people with PD [33,79,80]. Similarly, exercise may be an appropriate nonpharmacologic therapy in PD, as non-motor symptoms, depression [81], cognition [82], apathy, fatigue, and sleep are significantly improved [34,83]. Studies have confirmed that physical activity can delay the loss of motor skills and palliate non-motor symptoms, as well as increase quality of life in PD patients. In addition, exercise in older people has been shown to improve mood, elevate self-esteem and body image, and provide opportunities for social interaction [84].

Regarding the efficacy of the SSE in preventing falls and improving balance, our study involves a control group performing their “usual care”. It would be of great interest for future studies to investigate the additional value of SSE compared to other physical activities such as walking or any kind of sport that require balance control. If similar results between SSE and those sport or physical activities are found, then it will justify the use of SSE as an easy cost-effective and group applicable tool [78].

If the efficacy of this study is demonstrated, it would have a great impact on the HRQoL of people with PD because, as has been shown, it would favor the functional components of physical fitness as well as non-motor symptoms. At the same time, it would have a great impact in the socio-health field because it could be implemented in Parkinson’s associations, and even in the homes of people with PD for daily practice, with the help or supervision of the main caregivers, and could represent a great saving in the health system because it could reduce the number of falls and hospitalizations and also promote the autonomy of people with PD.

Finally, it should be pointed out that the progressive aging of the population leads us to expect an increase in PD because there is a close relationship between age and Parkinson’s disease. This growing reality will mean a greater social, economic, and health burden for public institutions and for society as a whole, so developing new rehabilitative therapies would help to meet the needs of people with PD and improve HRQoL.

Once positive results concerning the safety and applicability of the different physical tests and improved balance and reduction of falls have been observed, then proposals transferring the results to hospitals, health centers, and PD associations can be suggested. At the same time, positive results in psychological variables will help to design optimal physical exercise programs to improve the quality of life of these populations. Finally, if the interventions prove to be effective, this study would provide complementary actions to the usual care for the prevention of falls in people with PD.

The present manuscript has several limitations. Firstly, the study only investigates the time period of 8 weeks in people with Parkinson’s disease, but it would be interesting to know the effects of training in the medium and long term. Secondly, the objective intensity of the training cannot possibly be controlled through the evaluation of heart rate because we do not currently have adequate instruments to do so; only the intensity received will be evaluated through the modified Borg scale [85]. Third, the intervention will be carried out indoors. However, the scientific literature on outdoor interventions has shown that there is a greater benefit in some psychological aspects, as well as improving vitamin D synthesis [86]. Finally, a control group with specific balance training has not been included.

## 4. Conclusions

The present study will investigate the efficacy of SSE in people with PD for 8 weeks, with the aim of preventing falls and improving cognitive and psychological aspects. The results of this study will contribute to promoting the autonomy of people with PD and could reduce health care costs by reducing the number of falls. Likewise, if the efficacy of the therapy is demonstrated, it could be implemented in those centers, entities, and associations specialized in the care of people with PD.

## Figures and Tables

**Table 1:** Square Stepping Exercise training program.

Weeks	Days per Week	Difficulty	Number of Steps per Sequence	Duration	Auditory Stimulation (Metronome)
1	3	Beginner 1 and 2	4	50 min	+
2	3	Intermediate 1	6	50 min	
3	3	Intermediate 2	6	50 min	+
4	3	Intermediate 3	8	50 min	
5	3	Advanced 1	8	50 min	+
6	3	Advanced 2	8	50 min	
7	3	Advanced 3	8	50 min	+
8	3	Advanced 3	8	50 min	

**Table 2:** Schedule of experimental and control group evaluations.

Measures	Week 0	Week 2	Week 4	Week 6	Week 8
Applicability	+			+	+
Security	+			+	+
Number of falls, last 6 months and last year	+			+	+
Sociodemographic data	+				
Balance	+	+	+	+	+
Fear of falling	+	+	+	+	+
Physical condition	+	+		+	+
Health-related quality of life	+	+	+	+	+
Depression	+	+	+	+	+
Cognitive aspects	+	+	+	+	+
Perceived social support	+	+	+	+	+
Anticipatory cognition	+	+		+	+
